# Immune checkpoints: new insights into the pathogenesis of thyroid eye disease

**DOI:** 10.3389/fimmu.2024.1392956

**Published:** 2024-05-16

**Authors:** Xingyi Shu, Yuchao Shao, Yuqing Chen, Chengcheng Zeng, Xiao Huang, Ruili Wei

**Affiliations:** Department of Ophthalmology, Changzheng Hospital of Naval Medicine University, Shanghai, China

**Keywords:** thyroid eye disease, immune checkpoints, immune cells, teprotumumab, therapeutic targets

## Abstract

Thyroid eye disease (TED) is a disfiguring autoimmune disease characterized by changes in the orbital tissues and is caused by abnormal thyroid function or thyroid-related antibodies. It is the ocular manifestation of Graves’ disease. The expression of thyroid-stimulating hormone receptor (TSHR) and the insulin-like growth factor-1 receptor (IGF-1 R) on the cell membrane of orbital fibroblasts (OFs) is responsible for TED pathology. Excessive inflammation is caused when these receptors in the orbit are stimulated by autoantibodies. CD34^+^ fibrocytes, found in the peripheral blood and orbital tissues of patients with TED, express immune checkpoints (ICs) like MHC II, B7, and PD-L1, indicating their potential role in presenting antigens and regulating the immune response in TED pathogenesis. Immune checkpoint inhibitors (ICIs) have significantly transformed cancer treatment. However, it can also lead to the occurrence of TED in some instances, suggesting the abnormality of ICs in TED. This review will examine the overall pathogenic mechanism linked to the immune cells of TED and then discuss the latest research findings on the immunomodulatory role of ICs in the development and pathogenesis of TED. This will offer fresh perspectives on the study of pathogenesis and the identification of potential therapeutic targets.

## 1 Introduction

Immune checkpoint inhibitors (ICIs) represent groundbreaking progress in cancer therapy. ICIs can stimulate the immune system by blocking the ligands or receptors that impede the activation of T cells (1). This helps prevent tumor cells from evading the immune response and improves the body’s capacity to eliminate tumor cells. At present, the ICIs permitted by the FDA to treat cancer mainly target cytotoxic T-lymphocyte antigen 4 (CTLA-4), programmed cell death protein 1 (PD-1), and PD-ligand 1 (L1) (2). In addition, newly discovered immune checkpoints (ICs) such as lymphocyte-activation gene 3 (LAG3), T cell immunoglobulin and mucin domain 3 (Tim-3), as well as T cell immunoreceptor with immunoglobulin and ITIM domain (TIGIT) are also attracting attention in the field of cancer immunotherapy (3–6).

However, many patients receiving ICIs treatment in clinical practices exhibit clinical manifestations similar to autoimmune diseases, which are called immune-related adverse events (irAEs) (7). The irAEs can impact multiple systems in the body. One of the most prevalent typesof these irAEs is ICIs-induced endocrinopathies, which affect up to 40% of ICIs treated patients, with the thyroid being the most commonly affected endocrine organ (8).ICIs can also lead to various eye disorders such as optic neuritis, myasthenia gravis, Lambert-Eaton myasthenic syndrome, orbital myositis, internuclear ophthalmoplegia, opsoclonus-myoclonus-ataxia syndrome, oculomotor nerve palsy, uveitis, choroidal neovascularization, and thyroid eye disease (TED) (9–11). TED, also known as thyroid-associated ophthalmopathy (TAO) or Graves’ ophthalmopathy (GO), is an autoimmune inflammatory disease that occurs in the orbital tissues and is usually considered an extrathyroidal manifestation of Graves’ disease (GD), which is the most common cause of hyperthyroidism (12, 13). TED occurs in 25–50% of patients with GD (14). Some patients with chronic thyroiditis also develop TED (13). In some cases, TED can be found in patients with either hypothyroidism or normal thyroid function, which may be attributed to the gender and age of the patients (15, 16). However, according to the currently available case reports, most of the ICIs-treated patients who developed TED are euthyroid (**Table 1**). Whether this phenomenon indicates that ICs are involved in the orbital pathological changes in a way independent of GD pathogenesis remains elusive.

**Table 1:** Case reports of thyroid eye disease following immune checkpoint therapy.

ICIs	Dose	Symptoms	Thyroid function^a^	TRAb^a^	Imaging^a^	Reference
Ipilimumab	4 doses of ipilimumab at 10 mg/kg	severe eye pain proptosis periorbital edema	euthyroid	normal^b^	bilateral thickening of extraocular muscles	Min et al. (17)
Ipilimumab	12 doses of ipilimumab at 3 mg/kg	ophthalmoplegia bilateral proptosis	euthyroid	<0.3 IU/L	bilateral enlargement of all the extra-ocular muscles	McElnea et al. (18)
Ipilimumab	2 doses of ipilimumab	severe proptosis diplopia exposure keratitis	T3 slightly depressed^b^	46.9IU/L	enlargement of all extraocular muscles Crowding of the orbital apex	Borodic et al. (19)
Ipilimumab	3 doses of ipilimumab at 10 mg/kg	bilateral periorbital swelling upper eyelid retraction proptosis ophthalmoplegia	T4 8.56	<0.90 (neg)	symmetric enlargement and enhancement of the extraocular muscles	Sheldon et al. (20)
Tremelimumab	6 doses of tremelimumab at 10 mg/kg	acute periocular swelling erythema bilateral exophthalmos	hyperthyroidism	normal^b^	bilateral enlargement of all 4 extraocular rectus muscles with sparing of the tendons	Sagiv et al. (21)
Ipilimumab ceased; then switched to Pembrolizumab^c^	1 doses of ipilimumab at 10 mg/kg and 1 doses of pembrolizumab at 200mg	acute onset of proptosis chemosis diplopia decreased visual acuity of both eyes	euthyroid	33.64 IU/L after ipilimumab >40 IU/L after pembrolizumab	enlargement of bilateral inferior rectus muscles.	Rhea et al. (22)
Pembrolizumab	3 doses of pembrolizumab	progressive asymmetric proptosis new-onset diplopia eyelid retraction exposure keratopathy	euthyroid	–^b^	a prominent increase in orbital fat volume with secondary asymmetric exophthalmos mild tendon-sparing enlargement of the extraocular muscles	Park et al. (23)
Nivolumab	nivolumab at 3mg/kg^d^	bilateral upper eyelid retraction double vision limitation of eye movements	hyperthyroidism	elevated^b^	bilateral enlargement of the inferior and medial rectus muscles, with sparing of the tendons	Sagiv et al. (21)
Ipilimumab& Nivolumab	3 doses of ipilimumab at 1 mg/kg and nivolumab at 3 mg/kg	periocular pain pain with eye movement ocular irritation eyelid swelling and erythema double vision	euthyroid	normal^b^	bilateral symmetric enlargement of all extraocular muscles with sparing of the tendons	Sagiv et al. (21)

a. All data are post-treatment with immune checkpoint inhibitors.

b. Data not shown.

c. Development of Graves’ ophthalmopathy after treatment with ipilimumab and recurrence with pembrolizumab in a patient with previously treated Graves’ disease.

d. The patient had a history of GD.

The disruption of immune homeostasis significantly contributes to the pathogenesis of thyroid eye disease. The fundamental pathological process in the initial stages of TED involves the loss of T cell tolerance to the thyroid-stimulating hormone (TSH, or thyrotropin) receptor (TSHR), which occurs due to multiple factors (24). Of note, Covid-19 vaccination appears to increase the risk of TED development (25). After the loss of immune tolerance, B cells are activated through their interaction with helper T cells (Th), resulting in the production of autoantibodies. Stimulatory autoantibodies bind to and activate the TSHR and insulin-like growth factor-1 receptor (IGF-1R) expressed on the cell membrane of orbital fibroblasts (OFs), causing them to secrete various cytokines, which manifests orbital inflammation and differentiation of OFs into myofibroblasts or adipocytes (26). Patients with TED exhibit distinct phenotypic changes in immune cells of peripheral blood compared to normal individuals. These changes include abnormal proportions of CD4^+^ T cells and CD8^+^ T cells, an imbalance of Th1 and Th2 ratios (27), an increased proportion of Th17 cells (28), and a decrease in regulatory B cells (Breg) (29). Changes in the expression levels of various ICs, such as PD-1 and Tim-3, can also be detected in the immune cells of TED patients (30, 31). Additional investigation regarding the infiltration of immune cells in orbital tissues has the potential to enhance the understanding of the function of immune cells in the orbital inflammation associated with TED. The orbital tissues of individuals with TED contain a diverse array of infiltrating immune cells (32), and a positive correlation exists between the degree of infiltrating T and B cells with the clinical activity score (CAS) of TED patients (33). The infiltration of M1-like macrophages is more significant during the active phase, while M2-like macrophages dominate during the stable phase (34). While immune cells are not the primary cells mediating the pathological alterations in TED orbital tissues, research has shown a close interaction between immune cells and OFs (35). Therefore, exploring the difference between immune cells in the peripheral blood and orbital tissues of TED patients is essential and may help deepen our understanding of the interaction of immune cells and OFs.

The ICs have a crucial function in modulating the activation pathway of immune cells. They may either stimulate or inhibit immunological responses. ICs are essential for maintaining self-tolerance by controlling immune reaction type, magnitude, and duration (5). Different single nucleotide polymorphisms (SNPs) of ICs, including CTLA-4, HLA-DR3, and CD40, can be identified in patients with TED, indicating mutations in various ICs serve as an endogenous reason for TED development. Furthermore, examination of immune cells in the peripheral blood or orbital tissues of patients with TED can uncover atypical expression of PD-1, CTLA-4, and Tim-3. Interestingly, it has been found in recent years that CD34^+^ fibrocytes, which have a crucial role in the progression of TED, also constitutively express MHC II, B7, PD-L1 (36), and these molecules are significantly downregulated when exposed to teprotumumab (an IGF-1 inhibitor). A thorough investigation into ICs can greatly enhance the comprehension of the involvement of immune cells in TED and aid in the discovery of novel therapeutic targets.

## 2 Overview of thyroid eye disease

TED has been plaguing scientists and physicians around the world for nearly two centuries since its discovery (37). Histopathological features in the orbit of TED patients include immune cell infiltration and edema in the early stage, and tissue degeneration and fibrosis in the later stage (26). The most significantly affected areas are the extraocular muscles and orbital adipose tissue. Extraocular muscles and orbital adipose tissue are the most significantly involved. Based on the severity, TED can be divided into mild, moderate-to-severe, and sight-threatening, with the clinical activity score (CAS) serving as the best-validated scoring system to assess inflammation in the orbit, which is thus used to classify TED patients into active and inactive phases accordingly (13). Patients with TED commonly experience a wide range of clinical symptoms and signs. At the active phase, excessive inflammation in the orbit leads to periorbital tissue and conjunctival redness, swelling and congestion. Meanwhile, persistent enlargement of orbital adipose tissue and extraocular muscles results in proptosis. The secretion and accumulation of hyaluronic acid eventually cause fibrosis of the extraocular muscles and lead to diplopia and ocular motility disorders. Lid retraction, a characteristic manifestation of TED, stems from the involvement of levator palpebrae superioris and Müller muscle. In severe cases, corneal ulcers derived from incomplete eyelid closure and dysthyroid optic neuropathy (DON) may occur. DON is characterized by optic nerve compression due to orbital tissue proliferation, potentially causing blindness (26, 38).The primary non-surgical treatment for active moderate-to-severe TED is high-dose glucocorticoids (13). In clinical practice, patients often express significant concern about appearance changes caused by proptosis, which impose a heavy psychological burden on them. While glucocorticoids can alleviate ocular inflammation to some extent, their effectiveness in reducing proptosis is always limited (39). Additionally, their use is commonly accompanied by adverse effects such as drug-induced hepatotoxicity, glaucoma, hyperglycemia, gastric ulcers, and osteoporosis (40, 41). The efficacy of alternative immunomodulators like rituximab and tocilizumab in managing TED is still under investigation, with conflicting conclusions across different studies (8, 42–45). TED duration may be an important factor in their efficacy (8, 42). Notably, rituximab may increase the risk of DON (42, 46). Larger randomized controlled clinical trials are necessary to elucidate their effectiveness and safety in the treatment of TED. Teprotumumab represents a revolutionary breakthrough in TED treatment, demonstrating favorable effectiveness in phase II and III clinical trials (47, 48). Moreover, teprotumumab presents promising results in alleviating proptosis. However, its high price renders it inaccessible to many patients. Hence, it is imperative to investigate the development of TED and identify novel therapeutic targets.

## 3 Implication of immune cells in pathogenic mechanism of thyroid eye disease

In the initial stages of TED, a combination of genetic and environmental factors contribute to the development of intolerance to TSHR (39). Then, antigen-presenting cells (APC) can recognize, internalize, and degrade TSHR. They then present the self-antigen to Th through MHC II, activating Th. The Th interacts with B cells via the CD40L (CD154)-CD40 pathway. This interaction leads to the activation of B cells, which then secretes interleukin (IL) -2 and interferon (IFN) -γ. These cytokines promote the generation and release of self-antibodies against TSHR and IGF-IR, such as thyroid-stimulating hormone receptor antibodies (TRAb) and autoimmune IgGs (26).

OFs are the main culprit of pathological changes in TED orbital tissue. The expression of TSHR can be detected in normal OFs, which is significantly increased in TED-OF (49). The TSHR of OFs can be recognized and activated by autoantibodies generated by B cells. In addition, an increase was observed in the expression of IGF-1R on OFs in TED, which can also bind to autoantibodies found in TED patients. This binding process then triggers the activation of OFs (50). There is an interaction between the downstream signaling pathways of TSHR and IGF-1R. Both downstream signaling pathways exert their effects on nuclear Forkhead transcriptional factors (FOXOs), leading to the activation of FOXOs, which in turn reduces adipogenesis and hyaluronic acid synthesis in OFs (51, 52). Furthermore, IGF-1 can increase the expression of TSHR (53). Thus, the synergistic effect of TSHR and IGF-1R leads to the activation of OFs and the subsequent development of pathogenic alterations in the orbit. Two types of OFs can be distinguished based on the varying expressions of CD90 (Thy-1) and these two types have distinct differentiation orientations. Under the action of transforming growth factor (TGF) -β, CD90^+^ OFs differentiate into myofibroblasts and secrete hyaluronic acid, ultimately leading to fibrosis of the extraocular muscles in TED patients (54). Unlike CD90^+^ OFs, CD90^-^ OFs differentiate into adipocytes upon the activation of peroxisome-proliferator–activated receptor γ (PPAR-γ) which is a key regulatory element in adipogenesis (55). The variation in CD90 expression is one of the possible causes of various clinical symptoms observed in TED patients.

Upon activation, OFs secrete various chemokines, such as IL-6, monocyte chemoattractant protein-1 (MCP-1), Regulated upon Activation, Normal T Cell Expressed and Secreted (RANTES), IL-16, *etc.* (28, 56), which causes more immune cells in peripheral blood to migrate to the orbital tissues and exacerbate the local inflammatory response in the orbit. Alterations in immune cells can likewise be observed in the orbital tissues in individuals with TED. Single-cell sequencing results showed that the infiltration of immune cells in the orbital tissues of TED patients differed from that of healthy individuals (32). The proportion of natural killer (NK) cells, T cells, and myeloid cells was significantly increased in TED patients. A multicenter, single-blind case-control study using immunohistochemistry revealed a substantial increase in the level of lymphocyte infiltration in the orbital tissues of patients with active TED compared to both patients at stable phase and healthy controls (57). Another research revealed a notable rise in the expression of forkhead box protein P3(Foxp3)and CD40 in the orbital adipose tissue of individuals with TED, while the expression of CTLA-4 was found to be reduced. Additionally, the study revealed a positive correlation between disease severity and the expression levels of Foxp3 and CD40. Conversely, there was a negative correlation between disease severity and the expression of CTLA-4, CD28, and CD40L (58). The elevated expression of Foxp3 indicated that regulatory T cells (Tregs) could inhibit the immune response of individuals with TED, but the ability of Tregs to suppress inflammation is hindered. The dysfunction of Tregs may be associated with the reduction in the expression of CTLA-4, resulting in a decline in the inhibitory signal for T cell activation.

The direct or indirect interaction between OFs and immune cells infiltrating the orbital connective tissue, which is particularly significant between OFs and T cells, has been disclosed by many studies. The expression of CD40 is upregulated on OFs in TED and binds to CD154 on the surface of T cells. Thus, CFZ533, a monoclonal antibody targeting CD40, is considered a promising therapeutic agent for the treatment of TED (59). An *in vitro* experiment has shown that T cells activate OFs by secreting various cytokines, such as IFN-γ and tumor necrosis factor (TNF)-α (26). Feldon et al. found that activated T cells can secrete endogenous prostaglandins by upregulating the expression of cyclooxygenase-2 (COX-2), which serves as a ligand for PPAR-γ and activates lipidogenic differentiation in OFs (60). Additionally, there have been case reports indicating that the use of pioglitazone, a PPAR-γ agonist, might lead to cute exacerbation of symptoms in individuals with stable TED (61). However, PPAR-γ activation in immune cells, like lymphocytes and macrophages, can also have anti-inflammatory effects (62, 63). Given that the expression of CD90 is associated with the differentiating features of OFs, it is crucial to differentiate the involvement of PPAR-γ in CD90^+^ and CD90^-^ OFs. This distinction may have great value in investigating the therapeutic possibilities of PPAR-γ in TED.

In recent years, a group of CD34^+^ CXCR4^+^ Collagen 1^+^ cells has been identified in the OFs cultured from orbital adipose tissue of TED patients (64). These cells have comparable morphological features to OFs, but their surface markers differ from the orbital resident OFs, which often lack the CD34 marker. The CD34^+^ CXCR4^+^ Collagen 1^+^ cells are considered fibrocytes, which are monocyte-derived progenitor cells trafficking from bone marrow to the TED orbit (65). The proportion of CD34^+^ fibrocytes in the peripheral blood mononuclear cells (PBMC) of TED patients is significantly increased, and they highly express TSHR (66). These CD34^+^ fibrocytes can migrate and infiltrate into the orbital tissue, ultimately differentiating into CD34^-^ OFs that secrete Slit2 protein, contributing to the pathological changes in the orbits of TED patients (67). CD34^+^ fibrocytes in PBMC of patients with TED exhibit high MHCII and B7 expression. This enables CD34^+^ fibrocytes to deliver the two signals required for T cell activation. Consequently, CD34^+^ fibrocytes are recognized for their potent antigen-presenting capabilities (36). Further research is needed to fully understand the function of CD34^+^ CXCR4^+^ Collagen 1^+^ fibrocytes in peripheral blood and orbital tissues. Interfering with their direct interaction with the immune cells through ligand-receptor bridges or indirect interaction through cytokines could potentially provide new treatment strategies for TED.

## 4 Specific role of immune checkpoints in the pathogenesis of TED

**Figure 1:**
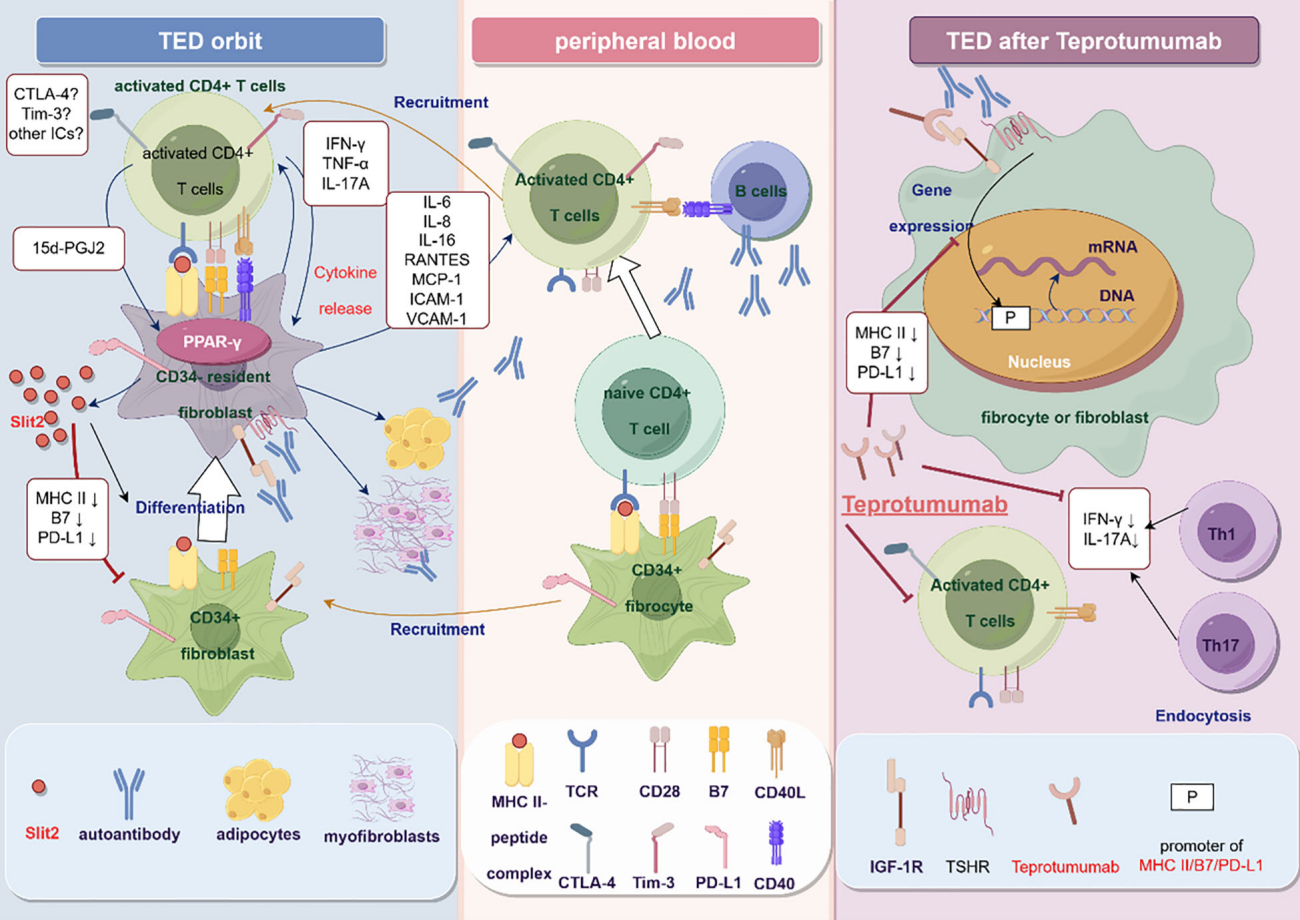
The potential mechanism of immune checkpoints in the pathogenesis of TED (By Figdraw). In peripheral blood, CD34^+^ fibrocytes with antigen-presenting function interact with and then activate naive CD4^+^ T cells, with the alteration of the expression of CTLA-4, Tim-3, and PD-1 on the activated T cells. Activated T cells activate B cells through the CD40-CD40L pathway and produce autoantibodies. Autoantibodies bind and activate TSHR and IGF-1R on the orbital fibroblasts, promoting the secretion of chemokines and recruiting T cells to the orbit, exacerbating local inflammation. T cells further activate orbital fibroblasts, causing them to differentiate into adipocytes and myofibroblasts. CD34^+^ fibrocytes also migrate to the orbit and differentiate into CD34^-^OFs under the action of Slit2 secreted by orbital fibroblasts *in situ*, accompanied by downregulation of immune checkpoint molecules such as MHC II, B7, and PD-L1. Teprotumumab can inhibit the interaction between T cells and OFs or fibroblasts by downregulating MHC II, B7, and PD-L1 molecules. Teprotumumab can also reduce inflammation by directly reducing the number of fibrocytes-adherent CD4^+^ T cells in peripheral blood and attenuating the secretion of IFN-γ and IL-17A.

The activation of naïve T cells requires simultaneous stimulation of two different extracellular signals. The first signal is the binding of the MHC-antigen peptide complex on the APC to the T-cell receptor (TCR), which then transmits activation signals to the cells *via* CD3. The second signal refers to the interaction between the co-stimulatory molecules and their corresponding ligands on the surface of the T cells. CD80 (B7.1) and CD86 (B7.2) enhance T cell activation by attaching to CD28 on the T cell. The APCs were found to play a role in the proliferation and differentiation of naïve T cells. Aside from B7 and CD28, there exist several co-stimulatory or co-inhibitory molecules on the APCs and T cells, which play a role in regulating the activity of APCs and T cells and maintaining the body’s immune homeostasis. The term used to refer to these molecules is immune checkpoints. Subsequent investigations have revealed that individuals with TED have alterations in the expression of several immunological checkpoints. Regulation of ICs and reinstating immune homeostasis in the body might be a viable approach for managing TED. In this section, we will summarize the research advance in the role of ICs in the pathogenesis of TED to date (**Figure 1**).

### 4.1 CD40/CD40L pathway

CD40 is a member of the TNF-α receptor superfamily and is found in several types of cells, including B cells and myeloid cells, where it is expressed constitutively. CD40L is expressed on activated T cells and is usually not detected in resting T cells. The CD40L expression is detectable in activated Th1, Th2, Th17, follicular helper T cells (Tfh), and Tregs. However, the expression of CD40L in Tregs is significantly reduced compared to Th. Moreover, CD40L induces CD40 trimerization and mediates downstream signaling pathways. The CD40/CD40L pathway plays a vital role in adaptive immunity. CD40 on B cells binding to CD40L facilitates B cells proliferation, activation, and antibody production. In the presence of other T cell activation signals (MHC-II and B7), CD40 can promote the proliferation and differentiation of T cell into specific types with the assistance of cytokines (68).

In patients with TED, the expression of CD40 on OFs is reported to be higher compared to healthy individuals. Additionally, under the stimulation of IFN-γ secreted by T cells, CD40 on OFs of TED patients gets significantly upregulated (69). CD40 activation exacerbates orbital inflammation and extraocular muscle fibrosis. It has been shown *in vitro* that CD40 activation leads to an increased synthesis of IL-6, IL-8, prostaglandin E2 (PGE2), MCP-1, intercellular adhesion molecule (ICAM)-1, vascular cell adhesion molecule (VCAM)-1, and E-selectin, and also results in increased secretion of hyaluronic acid (56, 70–72). Several variables are involved in the pathogenesis of TED via the CD40 pathway. Adipsin, a serine protease homolog, has been implicated in the CD40-mediated stimulation of OFs in individuals with TED. Adipocytes mainly release Adipsin, but macrophages and monocytes can also generate it. Adipsin is a vital adipokine and one of the components of complement factor D, participating in the pathogenesis of various autoimmune diseases. Activation of TED-OFs by CD40L or IGF-1 causes fibroblasts to differentiate into adipocytes, leading to increased synthesis and secretion of Adipsin. This further increases the expression of downstream cytokines, including IL-6, IL-8, PGE2, ICAM-1, and CCL2 (73). In addition, there is a notable elevation in the expression of sphingosine-1-phosphate (S1P) in the orbital tissues of patients with TED. S1P has been demonstrated to stimulate fibrosis, lipid synthesis, and the release of inflammatory cytokines in OFs of individuals with TED. Research has demonstrated that CD40 enhances the production of S1P in TED-OFs and facilitates T cell migration (74). This study proposed that lipid metabolism may regulatethe migration of T cells to orbital tissues and that controlling the activity of enzymes associated with S1P metabolism might be an effective strategy for managing inflammation in the orbit. Furthermore, the TSHR and CD40 expression levels on CD34^+^ fibrocytes in the peripheral blood of individuals with TED are markedly elevated. TSH and CD40L can upregulate IL-6, IL-8, and IL-12 expression of CD34^+^ fibrocytes obtained from the peripheral blood of patients with TED (75–77). The genetic polymorphism of CD40 also indicates its important role in the pathogenesis of TED. A meta-analysis revealed that the increased frequency of the T allele at the CD40 rs1883832 locus was associated with a reduced risk of developing TED (78). The mutation at the CD40 rs1883832 locus may reduce the transcription of CD40 mRNA and thus inhibit the activation of OFs (79).

The CD40/CD40L pathway mediates the interaction between activated T cells and OFs in TED. Feldon et al. demonstrated that the expression of CD40L on T cells is essential for the activation of OFs in individuals with TED (80). The binding of CD40 on OFs and CD40L (CD154) on T cells in TED patients leads to OFs proliferation, and blocking CD40 or CD40L significantly reduces OFs proliferation. Th17 cells in the peripheral blood of patients with TED can interact with OFs through the CD40-CD40L pathway. Additionally, they can stimulate the secretion of IL-17 and bind to the IL-17RA receptor on the cell membrane of OFs. This interaction activates the MAPK pathway, promoting RANTES secretion by OFs (28) and further promoting the migration of Th17 and Th1 cells to the orbit. In an *in vitro* model, The concurrent administration of IL-17 and CD40L resulted in a significant augmentation in the production and release of RANTES in OFs. However, the IL-17 alone did not demonstrate any noteworthy secretion of RANTES. This suggests that the activation of CD40 upstream is accountable for the response to IL-17. In addition to the activation of OFs by T cells, the CD40/CD40L pathway also plays a key role in the activation of T cells by OFs in TED patients, evidenced by TED-OFs promoting the proliferation of autologous T cells (81). The proportion of CD4^+^ CD28^-^ T cells in the peripheral blood of TED is reported to be significantly increased in comparison to normal individuals and is positively correlated with disease severity (82). CD40L may be crucial in activating T cells in TED or other autoimmune diseases when CD28, the second T cell activation signal, is absent. The activation of CD4^+^ CD28^-^ T cells is potentially reliant on the activation of CD40L. This is evident as inhibitory antibodies against CD40L considerably impede the activation of CD4^+^ CD28^-^ T cells (82). The precise mechanism of action of CD4^+^ CD28^-^ T cells, which contribute to orbital inflammation in TED, remains unclear. However, in the thyroid tissue of GD patients, activated CD4^+^ CD28^-^ T cells can secrete various inflammatory factors, promoting local tissue inflammation and the secretion of TRAb by B cellsleading to thyroid cell proliferation and tissue hyperplasia. Compared with CD4^+^ CD28^+^ T cells, CD4^+^ CD28^-^ T cells are insensitive to signals for cell apoptosis (83), suggesting that T cell apoptosis deficiency may be involved in the orbital inflammation of TED.

Ultimately, CD40 shows potential as a viable focus for addressing TED therapeutically. RNA aptamers specifically targeted CD40(CD40Apt) in TED mice, thereby significantly inhibiting the CD40-CD40L pathway. Moreover, CD40Apt reduced the expression of CD40, collagen I, TGF-β, and α-SMA in mice’s extraocular muscles and orbital fat tissue. Additionally, the aptamers effectively suppressed the Erk, p38, JNK, and NF-κB signaling pathways (84). The therapeutic effect of CFZ533 (CD40 monoclonal antibody) on TED still needs further research. However, CD40 is widely expressed in various cells throughout the body, including B cells, macrophages, dendritic cells (DC), platelets, endothelial cells, and epithelial cells (68). Close monitoring of systemic side effects is necessary when using therapies that target CD40.

### 4.2 CTLA-4

CTLA-4, also referred to as CD152, is mainly found in Tregs and activated T cells. Its principal function is to control the activation of T cells during the early stage. This procedure mostly takes place within the lymph nodes. In naïve T cells, CTLA-4 is present in intracellular vesicles. The binding of CD28 and CD80/CD86 causes CTLA-4 to migrate to the membrane of T cells. In comparison to CD28, CTLA-4 shows a higher affinity for B7. Therefore, CTLA-4 competitively binds to the ligands CD80 and CD86 on APCs, transmitting inhibitory signals for T cell activation and preventing their further proliferation (85). CTLA-4 inhibitors hinder the transmission of inhibitory signals from CTLA-4 to cells, stimulating the proliferation of T cells that target tumor antigens and strengthen the immune system’s ability to eliminate tumor cells (7).

Currently, ipilimumab and tremelimumab are FDA-approved CTLA-4 inhibitors (86). There have been reports of cancer patients developing Graves’ disease following therapy with ipilimumab or tremelimumab (87–89). Administration of CTLA-4 inhibitors, either alone or in combination with PD-1/PD-L1 inhibitors, can lead to acute exacerbation of pre-existing Graves’ disease in advanced melanoma patients (90, 91). An initial case of TED development after four doses of ipilimumab treatment was reported by *Min et al.*, with the patient presenting severe eye pain, proptosis, and periorbital edema. The CT and MRI scans revealed significant thickening of the extraocular muscles, but laboratory testing indicated a normal concentration of TRAb (17). Subsequently, McElnea, Borodic, Sheldon, and Sagiv independently documented instances of tumor patients who had no prior record of Graves’ disease developing TED after receiving ipilimumab therapy (18–21). These patients presented varying thyroid function and TRAb (**Table 1**). Rhea reported a case of a patient with Graves’ disease who had a sudden and rapid worsening of TED symptoms after receiving ipilimumab treatment. The patient’s periorbital edema improved significantly after discontinuing ipilimumab and undergoing glucocorticoid pulse therapy. However, there was a subsequent recurrence of TED while receiving pembrolizumab treatment (22). Sagiv also documented a case of a patient experiencing TED after receiving treatment with tremelimumab, a recently approved monoclonal antibody that targets CTLA-4. The patient exhibited notable thickening of the four rectus muscles, and abnormalities in thyroid function were reported (21). These case reports suggest that CTLA-4 may underlie the pathogenesis of TED.

Researchers have been intrigued by the association between the risk of TED development and the polymorphism in the CTLA-4 gene for many years. Vaidya et al. first discovered that the G-carrying genotype in the CTLA-4 A/G polymorphism is associated with an increased risk of TED and the frequency of the G allele is positively correlated with the severity of TED (92). There has been considerable research interest in the SNP49 in exon 1 of the CTLA-4 gene. However, the findings from different studies have yielded inconsistent conclusions (93). Studies on gene polymorphisms in various ethnic groups have shown that the G allele at SNP49 is associated with an increased risk of developing TED (94). The findings of a systematic review indicated that the A/G polymorphism at the SNP49 in exon 1 of the CTLA-4 gene has been linked to the development of TED in Europeans. However, this association does not appear to be statistically significant in Asians. Among Europeans, the GG genotype has been observed to significantly elevate the likelihood of developing TED. Additionally, individuals carrying the G allele face a notably higher risk than those with the A allele (95). The mechanism may rely on the fact that the G allele at the SNP49 in exon 1 of CTLA4 leads to the substitution of threonine (Thr) for alanine (Ala), which results in incorrect processing of the CTLA-4 in the endoplasmic reticulum. This causes a reduction in glycosylation efficiency and decreases the expression of CTLA-4 on the cell membrane (96). Subsequent studies have shown a negative correlation between the “TT” genotype frequency at the CTLA-4 rs733618 and TED. Haplotype analysis indicates that Crs733618Crs16840252 may potentially affect the risk of development of TED among patients with GD (14). The T allele at position -318 in the CTLA-4 promoter region is also speculated to increase the risk of TED in GD patients (94, 97).

The exact function of CTLA-4 in the development of TED still needs to be fully understood. The expression of Foxp3 (a vital transcription factor in Tregs differentiation and maturation) and CD40 is significantly increased in the orbital fat tissue of TED patients. In contrast, the expression level of CTLA-4 decreases significantly. According to the study, a positive correlation was found between the severity of the disease and the expression levels of Foxp3 and CD40. On the other hand, there is a negative correlation between the severity of the disease and the expression levels of CTLA-4, CD28, and CD40L (58). This indicates that Tregs infiltrate the orbital tissue of individuals with TED, but their ability to suppress inflammatory responses is impaired. The compromised function of Tregs may be associated with the reduced production of CTLA-4, which reduces the suppressive signal for T cell activation.

### 4.3 PD-1/PD-L1

Unlike CTLA-4, the inhibitory effect of PD-1 on T cells usually occurs in the peripheral tissues (98). Typically, following the TCR and MHC-antigen peptide complex become linked, the proximal signaling molecules of TCR undergo phosphorylation and trigger the activation of downstream PI3K-Akt-mTOR, Ras-MEK-ERK signaling pathways to relay signals that activate T cells. Tumor cells have aberrantly increased expression of PD-L1. Following the interaction between TCR and the MHC-antigen peptide complex, PD-L1 specifically binds to PD-1 on the surface of T cells. This binding triggers the phosphorylation of tyrosine in the intracellular domain of PD-1, which then recruits SHP-2 to the ITIM motif. As a result, the phosphorylation of proximal signaling molecules of TCR, such as Lck and ZAP-70, is blocked. This disruption leads to abnormal downstream activation signal transduction of T cells, ultimately causing abnormalities in the T cell cycle, gene transcription, cell metabolism, and epigenetics. Consequently, the recognition and killing of tumor cells by T cells are compromised (99).

**Table 1** shows that some cancer patients experienced TED due to the administration of PD-1 or PD-L1 inhibitors. Park et al. reported the first case of bilateral TED following the treatment with pembrolizumab (PD-1 inhibitor) (23). After treatment with pembrolizumab, the patient developed asymmetric exophthalmos and diplopia in both eyes; however, the thyroid function remained normal. For patients with pre-existing Graves’ disease, there are reports of the occurrence of TED after treatment with nivolumab (PD-1 inhibitor) (21). Research has furthermore discovered that the occurrence of hyperthyroidism and hypothyroidism in individuals with cancer who use PD-1 inhibitors is significantly higher compared to those who receive CTLA-4 inhibitors or PD-L1 inhibitors alone (100), suggesting that immune imbalance caused by PD-1 abnormalities may play an essential role in TED. Further investigation revealed a significant increase in the presence of PD-1 on the outer layer of CD4^+^ T cells and CD8^+^ T cells in the peripheral blood of individuals with TED. Adding PD-L1 to the co-culture system of T cells and OFs significantly inhibited the secretion of IFN-γ, IL-1β, TNF-α, and IL-2. Moreover, it significantly downregulated the CD40L of T cells. The downregulation of CD40L may have a therapeutic impact on TED by downregulating the activation of CD40 on the surface of OFs (31). This work elucidated the mechanism of regulation of the CD40/CD40L pathway in patients with TED and proposed that PD-L1 might serve as a promising therapeutic agent for the treatment of TED. Furthermore, there was a significant increase in the proportion of PD-1^+^ cells in CD4^+^ T, CD8^+^ T, and CD19^+^ B cells in the peripheral blood of patients with GD (101). However, the involvement of PD-1 on the surface of B cells, in developing TED is still understudied. The role of CD19^+^ PD-1^+^ B cells in the pathogenesis of TED and the disease diagnosis may have potential implications against the production of autoantibodies.

Recent research has indicated that patients with TED exhibited a significant increase in MHC II, B7, and PD-L1 expression on CD34^+^ fibrocytes. In clinical trials, moderate to severe TED patients treated with teprotumumab showed a substantial reduction in MHC II, B7, and PD-L1 expression. However, this effect was not observed in patients receiving a placebo (36). Since PD-L1 plays an inhibitory role in T cell activation, and MHCII and B7 are two important co-stimulatory molecules, this study suggests that MHC II and B7 may mask the immunosuppressive effect of PD-L1 on CD34^+^ fibrocytes in TED patients. The upregulation of PD-L1 may be an adaptive response to excessive immune activation. The study also found that the secretion of Slit2 by OFs significantly downregulated the expression of PD-L1 on CD34^+^ fibrocytes. Suppressing the expression of Slit2 in patients with TED resulted in a significant increase in the expression of PD-L1. These findings indicate that in the orbital microenvironment, Slit2 may intensify local inflammation by modifying the expression of immune checkpoints.

### 4.4 MHC II

The major histocompatibility complex (MHC), also referred to as human leukocyte antigen (HLA), has a role in the development of TED due to its gene polymorphism. HLA is divided into type I (HLA-A, B, C) and type II (DP, DQ, DR). Type II HLA, or MHC II, is usually expressed on the surface of APCs, providing the first signal for CD4^+^ T cells activation, and can enhance the inflammatory response. In particular, MHC II is also expressed in the OFs and is essential for stimulating T cells in individuals with TED (80). The presence of TED increases MHC II in CD34^+^ fibrocytes. However, when IGF-IR inhibitors (teprotumumab, lisitinib, 1H7) or IGF-IR knockdownare used, the expression levels of MHC II decrease. This decrease is accompanied by a reduction in the number of CD4^+^ T cells adhering to CD34^+^ fibrocytes and a decrease in the secretion of IL-17A, which is mainly secreted by Th17 cells. These findings suggest that IGF-IR inhibitors may restore immune tolerance in TED patients by regulating the expression of immune checkpoints associated with T cell activation in CD34^+^ fibrocytes and influencing the proliferation and differentiation of CD4^+^ T cells (36). The presence of MHC II on OFs and fibrocytes, rather than MHC I, suggests their close direct interaction with CD4^+^ T cells in the pathogenesis of TED. Similarly, numerous studies have reported that the variation in type II HLA genes is linked to a higher risk of developing TED (102). However, there is less research on the differences in type I HLA variation between TED patients and healthy individuals, indicating that the CD4^+^ T cell subset may significantly impact the progression of TED.

### 4.5 Tim-3

The expression of Tim-3 is increased on the activated CD4^+^ T cells. Activation of Tim-3 suppresses the proliferation of T cells and the secretion of cytokines, leading to a decrease in the body’s inflammatory response and the preservation of peripheral immune tolerance. Research has demonstrated that individuals with TED exhibit significant reductions in the expression of Tim-3 on Th1 and Th17 cells in their peripheral blood in comparison to patients with GD. Furthermore, there is an inverse relationship between the number of Th1 and Th17 cells in the peripheral blood of TED patients and the proportion of Tim-3^+^ cells (30). This suggests that the downregulation of Tim-3 may disrupt immune tolerance in the body, increasing the immune activity of Th1 and Th17 cells and causing excessive local inflammation in the orbit region. Furthermore, research has shown that the presence of Gal-9 (the ligand for Tim-3) on DC cells in the peripheral blood of patients with TED is significantly reduced, and its expression is inversely associated with CAS. Adding Gal-9 to a co-culture system of peripheral blood DCs and lymphocytes significantly inhibits the secretion of Th1, Th2, and Th17-related cytokines. These studies reveal that dysfunction of the Gal-9/Tim-3 pathway may be associated with the pathogenesis of TED (103).

### 4.6 B7/CD28

B7.1 (CD80) and B7.2 (CD86) molecules are co-stimulatory molecules on the surface of APCs. These molecules competitively interact with CD28 against CTLA-4. The interaction between B7 and CD28 serves as the second signal in the T cell activation pathway, resulting in the proliferation, and differentiation of T cells, along with the release of downstream inflammatory cytokines (104). In patients with GD, the polymorphism of CD80 and CD86 genes is related to the risk of TED. Multifactorial dimensionality reduction analysis revealed that the interaction between CD80-rs9289131 and CD86-rs9872483 has a protective effect on the onset of TED (104). A study revealed that B7 molecules were consistently expressed on CD34^+^ fibrocytes, and the expression of B7 molecules may be significantly increased by thyrotropin or M22 (an antibody that stimulates the TSHR). Conversely, Slit2 decreases the expression of B7 molecules. These findings indicate that OFs regulate the expression of B7 molecules on CD34^+^ fibrocytes,which significantly impact T cell activation.

The mRNA levels of CD28 in the orbital tissues of severe TED patients are significantly lower than those in mild TED patients. Nevertheless, the degree of downregulation is less pronounced compared to CTLA-4 (58), indicating that the equilibrium between CD28 and CTLA-4 plays a role in preserving the immune homeostasis of the orbit.

### 4.7 CD52

CD52 is widely expressed on mature B and T cells, with relatively low expression on NK cells and other leukocytes. CD52 is not expressed in hematopoietic stem cells (105). Alemtuzumab is a monoclonal antibody that specifically inhibits the activity of CD52. The FDA has approved it for treating multiple sclerosis and B-cell chronic lymphocytic leukemia. Alemtuzumab primarily works by quickly reducing the number of mature lymphocytes in the bloodstream and then using the regeneration ability of hematopoietic stem cells to restore a balanced immune system in the body. A subset of multiple sclerosis patients developed TED as a consequence of taking alemtuzumab therapy (105–108). Presently, there is a lack of research on the involvement of CD52 in the development of TED. During immune system reconstitution, lymphocytes undergo rapid proliferation and sustained activation. This can lead to an imbalance in the ratio of Tregs to Th1 and Th2 cells, thereby exacerbating the body’s inflammatory response and stimulating the production of autoantibodies (109). Furthermore, following the administration of alemtuzumab, there was a significant increase in the levels of IL-21 in the peripheral blood of multiple sclerosis patients who had recently developed autoimmune diseases. The activation of the IL-21 receptor on effector T cells can stimulate the production of autoreactive T cells, potentially facilitated by IL-21 (110).

## Discussion and prospection

There are still some limitations in the research on the phenotype and function of immune cells in TED. Primarily, most research identifies immune cells in the peripheral blood, disregarding the presence of infiltrating immune cells in orbital tissues. Recent studies have shown the presence of tissue-resident immune cells in peripheral tissues, indicating that they settle in these tissues and are not circulatory in the bloodstream (111). Tissue-resident T cells and macrophages in the visceral adipose tissue regulate adipose tissue inflammation and lipid metabolism and thus maintain metabolic homeostasis (112, 113). Dermatomyositis and autoimmune enteropathy, among other autoimmune diseases, have discernible disparities in tissue-resident memory T cells compared with those without these conditions. A study utilized spatial transcriptomics to compare the expression of inhibitory T cell ICs, specifically CTLA-4, TIGIT, LAG-3, and PDCD1 (encoding PD-1), on tissue-resident memory T cells in patients with dermatomyositis (Th1-driven) and psoriasis (Th17-driven). The study revealed a significant increase in the expression of inhibitory T cell ICs on tissue-resident memory T cells in patients with dermatomyositis. This suggests that tissue-resident memory T cells may play a role in developing autoimmune diseases (114). Secondly, immune cells in tissues usually coordinate with different local environments. The orbital microenvironment contains many types of cells, including adipocytes, myocytes, and OFs. These cells can influence the activity of immune cells through interactions between cells and the secretion of cytokines. Hence, it is crucial to examine the phenotypic and functionality of immune cells inside the orbital tissues, together with their interaction with other cells in the orbit, to gain a deeper comprehension of the pathophysiology of TED.

The role of immune checkpoints in regulating the development of TED appears to be specific since not all co-inhibitory immune checkpoints that suppress immune responses exhibit identical patterns of change in TED. For instance, the expression of PD-1 is increased in the peripheral blood of T cells, while the expression of Tim-3 is decreased in TED patients. Additionally, the expression of PD-L1 is increased in fibrocytes. In the orbital tissues of severe TED patients, CTLA-4 expression is significantly lower than in those of mild patients. Therefore, it is necessary to study the regulatory role of a single immune checkpoint in the pathogenesis of TED. The metabolism of immune cells plays a crucial role in controlling the differentiation of immune cells, the expression of immune checkpoints, and the regulation of inflammation in tissues. In visceral adipose tissue, the activation of PPAR-γ in tissue-resident Tregs can effectively alleviate low-grade inflammation in adipose tissue (113), possibly by regulating T cell lipid metabolism mediated by CD36 and carnitine palmityl transferase1 (CPT)-1. This ultimately upregulates the expression of Foxp3 and inhibitory immune checkpoints CTLA-4 and TIGIT in T cells (115). Prior research has established that endogenous prostaglandins released by T cells modulate the activation of PPAR-γ in OFs (80). Further investigations on the immune metabolism of different immune cells in orbital tissues, as well as the regulatory function of OFs and CD34^+^ fibrocytes in immune metabolism, can enhance the understanding of the development and pathogenesis of TED.

In conclusion, the pathogenesis of TED is still not fully understood. Patients with TED often experience disfigurement, which brings a tremendous psychological burden to them. While the FDA has approved the use of teprotumumab in the treatment of TED, some patients may exhibit intolerance to this medication, experiencing adverse effects such as muscular spasms, nausea, and hearing impairment (47, 48, 116, 117), and the high cost of teprotumumab also brings heavy economic burden to patients around the world. The identification of CD34^+^ fibrocytes and the presence of immune checkpoints have introduced novel concepts in the investigation of the underlying processes of TED. The examination of the immune cells and immune checkpoints present in peripheral blood and orbital tissues can enhance the understanding of the disease and facilitate the identification of reliable biomarkers and novel therapeutic targets for clinical use.

## Author contributions

X-YS: Writing – original draft, Writing – review & editing. Y-CS: Writing – original draft, Writing – review & editing. Y-QC: Writing – review & editing. C-CZ: Writing – review & editing. XH: Validation, Writing – review & editing. R-LW: Validation, Writing – review & editing.
